# Adaptation of a school-based mental health literacy curriculum: from Canadian to English classrooms

**DOI:** 10.1017/gmh.2021.38

**Published:** 2021-10-12

**Authors:** Rosie Mansfield, Neil Humphrey, Praveetha Patalay, Anna Moore, Emily Stapley

**Affiliations:** 1 Centre for Longitudinal Studies, University College London, London, UK; 2 Manchester Institute of Education, University of Manchester, Manchester, UK; 3 MRC Unit for Lifelong Health and Aging, University College London, London, UK; 4 Evidence Based Practice Unit, Anna Freud National Centre for Children and Families, London, UK

**Keywords:** cultural adaptation, mental health literacy curriculum, school staff

## Abstract

**Background:**

School-based mental health literacy (MHL) interventions are increasingly trialled outside of the country in which they were developed. However, there is a lack of published studies that qualitatively explore their cultural adaptation. This study investigated the reasons for adaptations made and suggested to a Canadian MHL curriculum (The Guide) within the English school context.

**Method:**

Semi-structured interviews were conducted with 11 school staff responsible for the planning and/or implementation of The Guide across three schools in the South East of England, as part of the Education for Wellbeing (EfW) feasibility study. Transcripts were analysed using a hybrid, deductive-inductive thematic analysis.

**Results:**

Adaptations made and suggested included dropping and emphasising content, and adapting language, examples and references. Most adaptations were proactive and related to The Guide's implementation methods, including developing more interactive and student-led approaches. *Staff Capacity and Expertise*, *Timetabling,* and *Accessibility of Resources* were identified as logistical reasons for adaptations. Philosophical reasons included *Consistency of Messages*, *Student Characteristics*, *Reducing Stigma and Empowering Students*, *National and Local Context,* and *Appropriate Pedagogic Practices*.

**Conclusion:**

Overall, recommendations were for immediately implementable lesson plans informed by teachers' knowledge about best pedagogic practices in England. Adequate training, attended by both senior leadership and those implementing, was also emphasised. While ensuring that the core components are clear, MHL interventions should be developed with a necessary level of flexibility to accommodate contextual characteristics. Future research should ensure that adaptations are captured through process and implementation evaluations conducted alongside efficacy trials.

## Introduction

Adolescence is increasingly recognised as a key period for improving mental health literacy (MHL) and promoting access to services (O'Connell *et al*., 2009), with schools identified as a universal point of access (Fazel *et al*., 2014; Greenberg *et al*., 2017). Kutcher *et al*. (2016) defined MHL as: ‘*(1) understanding how to obtain and maintain positive mental health, (2) understanding mental disorders and their treatments, (3) decreasing stigma related to mental disorders, and (4) enhancing help-seeking efficacy (knowing when and where to seek help and developing competencies designed to improve one's mental health care and self-management capabilities)*’ (p.155).

Based on the above definition, The Guide was developed in Canada for adolescents aged 13–15. The original web-based curriculum guide aimed to increase students' MHL and consisted of six evidence-based modules. The Guide was first trialled in Canada and delivered by teachers in grade 9 (ages 14–15) health classes. Multiple pre-post follow-up studies have shown significant improvements in teacher and student knowledge and attitudes relating to mental illness following The Guide curriculum, and have indicated some sustained effects (Kutcher *et al*., 2013, 2015; Mcluckie *et al*., 2014). Furthermore, in a randomised controlled trial (RCT) investigating the impact of The Guide curriculum for grade 11 and 12 students (ages 16–18), significant improvements in knowledge and attitudes were found (Milin *et al*., 2016). The Guide has also been translated, adapted and trialled outside of Canada, showing improved MHL outcomes for teachers and students in Tanzania, Malawi (Kutcher *et al*., 2015, 2016, 2017) and Nicaragua (Ravindran *et al*., 2018).

Despite an increased number of RCTs in recent years, reviews of school-based MHL interventions and universal, mental health awareness programmes conclude that more research is needed to confirm their effectiveness, alongside a greater focus on strengths and weaknesses of interventions for successful implementation (Wei *et al*., 2013; Salerno, 2016). Few evaluations of MHL interventions have conducted implementation and process evaluations, with researchers often only focusing on fidelity (e.g. Chisholm *et al*., 2016). With the increased transportation of MHL interventions across countries, it is critical to explore the processes by which interventions are adapted, and to document what adaptations are made, when, why and by whom. Documenting these processes can inform the development of flexible, appropriate and feasible approaches to MHL promotion while maintaining clear logic models, striking a balance between required fidelity and necessary cultural adaptations (Ferrer-Wreder *et al*., 2012).

The success of imported preventive interventions is somewhat dependent on their level of adaptability, with the view that adaptations are inevitable in real-world settings (Castro *et al*., 2004; Carvalho *et al*., 2013; Moore *et al*., 2013). While surface-level adaptations are generally expected and may improve the cultural fit of an intervention, some argue that adaptations create uncertainty and threaten their potential effectiveness (Elliott and Mihalic, 2004). This is known as the fidelity-adaptation dilemma (Castro *et al*., 2010). Given that programmes can produce positive outcomes with as little as 60% fidelity (Durlak and DuPre, 2008), it is clear that the quality and valence of adaptations are just as important for intervention efficacy (Hansen *et al*., 2013; Humphrey *et al*., 2018).

Despite increased demand for transportation and adaptation of evidence-based interventions (Castro *et al*., 2010), there remains a lack of translational research that explores the specific structures and processes that determine intervention adaptation and implementation in practice (Spoth *et al*., 2013). Realist approaches to programme evaluation move beyond theoretically driven mechanisms through which interventions produce positive change, and acknowledge the interaction with context, and the dynamic and complex nature of these social systems (Lacouture *et al*., 2015). They seek to understand how the intervention works but also for whom and under what circumstances? (Pawson and Tilley, 2004). The importance of understanding not only the type of adaptations made, but also the knowledge and reasoning that informs them, is increasingly recognised (Humphrey *et al*., 2016). Examples of studies that report on the reasons for adaptations made to school-based interventions, and the process of cultural adaptation, are emerging in the areas of substance misuse prevention (e.g. Miller-Day *et al*., 2013; Marsiglia *et al*., 2019) and social and emotional learning (SEL) (Lendrum and Askell-Williams, 2019). However, to our knowledge, there are not yet any published articles that qualitatively investigate the cultural adaptation of a school-based MHL intervention.

### Aims and research questions

The aim of the current study was to explore the cultural adaptation of a Canadian MHL curriculum (The Guide) for delivery in English classrooms, by investigating the reasons for adaptations made and suggested by school staff involved in a feasibility study. The research question was: When trialling the feasibility of a Canadian MHL curriculum (The Guide) in England, what adaptations were made within the school context, when, why and by whom, and what adaptations were suggested for the future?

## Method

### The mental health and high school curriculum guide (The Guide)

The original Guide was a web-based curriculum resource developed in Canada for adolescents aged 13–15. The aim was to increase students' MHL through six evidence-based modules covering the core components of MHL. Each module included a full PowerPoint presentation with activities, and The Guide website provided additional materials such as teacher study resources. Modules were designed to be taught by class teachers and delivered over 10–12 hours, with each lesson scheduled for approximately 60 minutes. The mechanism through which The Guide aimed to improve student outcomes was increased teacher MHL.

### Participants and procedure

The interviews analysed in this paper formed part of the AWARE feasibility study (Approaches for Wellbeing and Mental Health Literacy: Research in Education), one of two parallel group clustered RCTs conducted by the Department for Education (England) funded Education for Wellbeing Programme (Hayes *et al*., 2019). AWARE is a three-arm, parallel group clustered RCT with the aim of comparing The Guide and Youth Aware of Mental Health (YAM), to a usual provision (control) condition. Schools expressed interest in the feasibility study via an online form and four schools in the South East of England were allocated to deliver The Guide in 2018 to approximately 90 students in years 9–10 (ages 13–15). Selected staff attended a teacher training day in February 2018 where schools were invited to become case studies. One-to-one interviews with school staff responsible for the planning and/or implementation were conducted mid to late delivery period as part of the case study visits.

The current study focused on two self-selected case study schools that implemented The Guide (Sc1 and Sc3), and one school that chose to drop out following the training (Sc2). The inclusion of staff interviews from the drop-out school provided a point of comparison in terms of reasons for not implementing *vs*. reasons for adaptations made and suggested to The Guide. Fully informed consent was gained from school staff on the day by a researcher. Eleven school staff were interviewed in April and May 2018. For an overview of school and participant numbers, and the method by which they were interviewed, see Table 1. Of those participants that provided demographic information, all identified as female (*n* = 8) and were between 28 and 52 years old (*n* = 5); three reported their ethnicity as Black and four as White (*n* = 7). Participants' roles within school varied [Senior Leadership Team *n* = 3, specialist role, e.g. Personal Social Health Education (PSHE) Lead *n* = 5, and classroom teacher *n* = 3]. For this reason, participants are referred to as ‘school staff’ throughout. Roles and responsibilities within school and in relation to The Guide are referred to in the results section including when adaptations were made and by whom?

**Table 1.** Overview of schools, participants and interview methods

School (Sc)	Participant (P)	Data collection method	Researcher
1	1	Face-to-face interview	RM
1	2	Face-to-face interview	RM
1	3	Face-to-face interview	ES
1	4	Face-to-face interview	ES
2	5	Phone interview	RM
2	6	Phone interview	RM
3	7	Face-to-face interview	RM
3	8	Face-to-face interview	RM
3	9	Face-to-face interview	RM
3	10	Face-to-face interview	AM
3	11	Face-to-face interview	AM

### Data collection

All interviews with school staff were conducted one-to-one either in a private room at the school or over the phone (drop-out school only). A semi-structured approach was adopted for interviews. This allowed for questions to guide specific topics of interest, while allowing flexibility for participants to offer new experiences and perspectives (Galletta, 2013). Interview schedules for schools that delivered The Guide included questions relating to opinions on the content and structure of The Guide for the English context and suggested improvements, experiences of implementing, including any adaptations made and why, and the perceived impact for students. Similarly, for the school that chose not to deliver The Guide, interviews explored opinions on The Guide, reasons for not implementing, and any suggested improvements to the materials and delivery methods. The mean interview length in minutes for school staff was *M* = 29.37 (s.d. = 9.00).

### Data analysis

A hybrid, deductive-inductive thematic analysis was conducted at a semantic level using NVivo 12 and followed Braun and Clarke's six-step approach (Braun and Clarke, 2006). The current study aimed to conduct an in-depth exploration of the adaptation process across a small number of schools. Applying statistical-probabilistic generalisability was therefore not appropriate. Instead, hybrid thematic analysis allowed a balance between applying existing theoretical frameworks to understand the generalisability of findings, and a more data-driven analysis of the unique experiences of school staff adapting and implementing a MHL intervention in the English school context.

The first (RM), fourth (AM) and last authors (ES), as well as members of the wider EfW team, familiarised themselves with the data by checking the accuracy of the transcription against the original audio files. RM led on all the remaining steps of the analysis. Two stages of coding were conducted. Firstly, the following deductive codes were developed in line with the research question: what, when, why, by whom and suggested improvements. Within these codes, additional deductive codes were identified using *a priori* themes from pre-existing adaptation and teacher knowledge theory (see Table 2 for *a priori* themes). These codes were refined during the first round to avoid too much overlap across codes (e.g. where theories had overlapping themes) and to include only codes relevant to the current data. For example, at this stage, the last author (ES) reviewed a sub-set of deductive codes relating to deep *v*. surface-level content adaptations to help inform the inclusion/exclusion of these codes.

**Table 2.** Overview of deductive codes and underpinning adaptation and teacher knowledge theory

Deductive codes: research question	Deductive codes: *a priori* theory	Underpinning theory	Reviewed deductive codes
What (adaptations made and suggested)	Content People Context Concepts Goals Metaphors Language Implementation methods Surface level Deep level	These codes were informed by the ecological validity model (EVM; Bernal *et al*., 1995; Bernal *et al*., 2009) and the cultural sensitivity model (CSM; Resnicow *et al*., 1999; Resnicow *et al*., 2000). Both models recommend cultural adaptations across content and implementation methods, and include changes to language, metaphors, concepts, people, context and goals. The CSM presents two types of adaptation: surface level or visible adaptations and deep, non-visible adaptations. By working to create an intervention sensitive to observable cultural characteristics such as language, clothes and names, as well as incorporating deeper, less visible cultural norms such as attitudes and behaviours that are influenced by historical, environmental, social and psychological factors, both models aim to increase the audience's engagement and enhance programme efficacy.	Content People Context Concepts Language Implementation methods
When	Proactive Reactive	The timings of adaptations were presented by Moore *et al*. (2013) and later by Humphrey *et al*. (2016). They define proactive adaptations as those changes made prior to implementation, in anticipation of a lack of fit to the context. In contrast, reactive adaptations are defined as those made in response to issues that arise during implementation.	Proactive Reactive
Why	Logistical reasons Philosophical reasons	Reasons for adaptations have also been split into logistical and philosophical. Logistical adaptations are defined as those made due to issues of time, capacity and resources, whereas philosophical adaptations relate to the perceived fit of an intervention in terms of how it aligns with the views and culture of the target population, and the organisations and individuals implementing it (Moore *et al*., 2013; Humphrey *et al*., 2016).	Logistical reasons Philosophical reasons
Why	Teachers' knowledge Content knowledge Curriculum knowledge General pedagogic knowledge Pedagogic content knowledge Knowledge of educational ends, purposes, values, philosophy, and history Knowledge of educational contexts Knowledge of learners and their characteristics	Lendrum and Askell-Williams (2019) applied Shulman's (1986, 1987) categories of teachers' knowledge to their analysis of adaptations made to the PATHS curriculum in England. Shulman used the following definitions for the different types of teacher knowledge. Content knowledge: refers to the specific intervention subject knowledge. Curriculum knowledge: refers to an understanding and awareness of available curriculum materials and interventions relating to a specific subject. General pedagogic knowledge: refers to general teaching practices, classroom organisation and time management of planning and timetabling. Pedagogic content knowledge: is a combination of specific subject knowledge with appropriate pedagogic practices. Knowledge of educational ends, purposes, values, philosophy and history: includes an understanding of the overall goals of the school, e.g. school ethos. Knowledge of educational contexts: refers to awareness and understanding of the school dynamic and governance as well as the local community context. Knowledge of learners and their characteristics: refers to an awareness of students' learning styles and teachers' knowledge of the characteristics of their class.	Teachers' knowledge Content knowledge Curriculum knowledge General pedagogic knowledge Pedagogic content knowledge Knowledge of educational ends, purposes, values, philosophy, history Knowledge of educational contexts Knowledge of learners and their characteristics
Why	Recipient group characteristics Characteristics of staff Administrative/community factors	Castro *et al*. (2004) describe a ‘cultural mismatch’ when a programme or intervention conflicts with the needs of the relevant populations in the adopting country or context. They present three potential sources of mismatch: group characteristics, delivery staff and administrative/community factors. Examples of group characteristics include language and ethnicity; staff characteristics include relevant skills and perspectives, and administrative and community factors include the organisation and infrastructure to implement the intervention.	Absorbed into logistical and philosophical codes and teacher knowledge
By whom	Characteristics of staff	As presented above, one potential source of cultural mismatch presented by Castro *et al*. (2004) is the skills and perspectives of the staff responsible for the planning and/or implementation of the intervention.	Characteristics of staff

Inductive coding was then conducted to identify codes specific to The Guide and the English school context, and the unique experiences of the school staff. A process of reorganising and combining codes resulted in a preliminary set of themes. These themes were reviewed by the lead author (RM) by ensuring the content of coded transcript extracts accurately represented the themes, and that all data relevant to the research question were adequately captured. This was an iterative process that continued through writing up themes and producing the final thematic map. A summary of data captured by each theme was written to form the narrative of the results section. The names and descriptions of themes as well as the selected data extracts were also reviewed in several discussions between the lead author (RM) and the fourth (AM) and last author (ES), to ensure that themes were accurately representing the data. A final review of results was conducted by all authors.

## Results

**Fig. 1.**
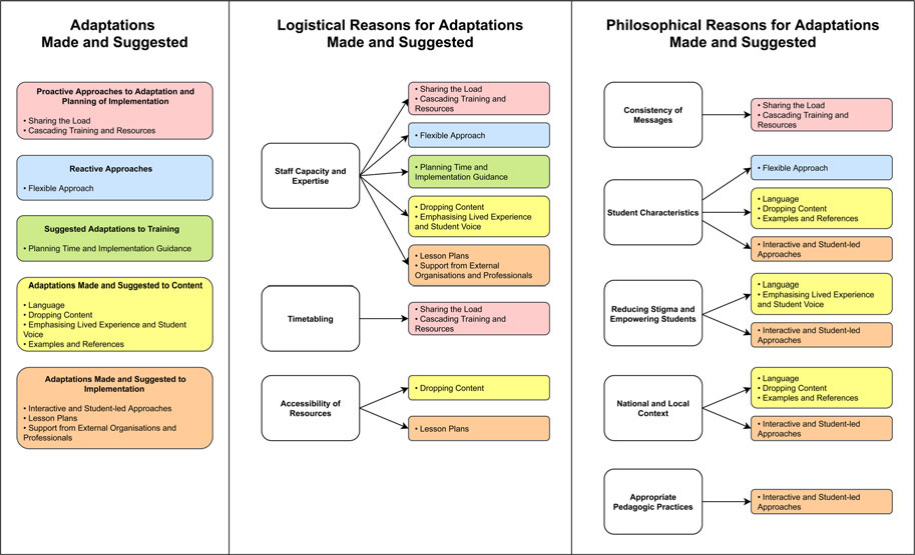
Thematic map of adaptations made and suggested and the logistical and philosophical reasoning.

Figure 1 presents a thematic map of logistical and philosophical reasons for adaptations made and suggested. These are linked to the different approaches to adaptation and implementation adopted by schools, as well as the types of adaptations made and suggested to The Guide's content and implementation. Sub-themes are presented as sub-headings in italics and are organised by higher-order, deductive themes.

### Overall perception of The Guide

Across the two schools that implemented The Guide, staff were generally positive about the content and viewed it as a quality assured set of resources. They valued the volume of information and materials provided within The Guide, which offered a ‘*one-stop shop*’ (Sc3, P7), and a level of flexibility to pick and choose content and activities relevant to their students. However, these comments were often caveated with the fact that, prior to implementation, there was a job for someone within school to translate the content into discrete lessons that could be delivered with consistency.

‘*My initial impression was there was a lot of content there which was great … that was kind of ideal to have something – it is all there, you know it is, kind of, a trusted resource, you‘ve got the video links and so on. So that, for me, was a real positive. The negative was that, as it stands, it is not deliverable in schools. Certainly not in our context.*’ (Sc3, P7)

Similarly, for the school that chose not to implement The Guide (Sc2), staff felt that a considerable amount of work was required to adapt materials and delivery methods to ensure they met the desired aims within their school context.

### When were adaptations made and by whom?

#### Proactive approaches

In line with schools reporting that a process of adaptation was necessary prior to implementation, the majority of changes reported were proactive, made prior to implementation in anticipation of a lack of fit. Different proactive approaches to adapting materials and planning the implementation were adopted.

***Sharing the load:*** For Sc1, the six modules were split so that staff were only responsible for the planning and implementation of one session. This approach also meant that sessions were delivered to larger groups of students (combined classes) in the school hall. All members of staff involved in implementation attended training and held specialist roles within the school [e.g. Special Educational Needs Coordinator (SENCO), Behaviour Manager, Safeguarding Lead]; the first two sessions were delivered by members of the Senior Leadership Team, and the Wellbeing Manager was present in all sessions.

‘*We've kind of broke it up into modules. So, we've each taken a module to design and to teach to the year group.*’ (Sc1, P4)

***Cascading training and resources:*** At Sc3, two members of staff attended The Guide training, adapted the materials and planned each module before cascading training to their colleagues (classroom teachers) involved in implementation. Resources were sent to these staff in advance of the timetabled sessions to ensure that they had enough time to familiarise themselves with the content. One member of staff who planned the modules was a member of the Senior Leadership Team, and both staff held specialist roles within the school (e.g. PSHE Lead, SENCO, Mental Health Lead). Modules were delivered by classroom teachers in tutor time as part of the PSHE curriculum.

‘*So, myself and (staff member) who came on the training, we sat down and kind of went through it and thought about, okay, what might be, what are the kind of key things you want to take from each module? What might be some activities that you could do? … What we tried to do is send it to people two or three weeks before they were delivering it, so they had time to digest and to go through it.*’ (Sc3, P11)

#### Reactive approaches

Fewer reactive adaptations were made within the sessions, and although some school staff reported making adaptations due to running out of time, most were in response to student engagement and could be organised into one theme.

***Flexible approach*:** Reactive adaptations related to a more flexible and organic approach that allowed the focus of the sessions to be led by students' interests.

‘*Rather than kind of bombard them with information on a PowerPoint I'd rather kind of pick things and just kind of like, you know, see what they're interested in, seeing what … seeing what's, you know, what's making their lights go on.*’ (Sc3, P8)

School staff also reported simplifying and translating content to make it more accessible. More reactive adaptations were reported by school staff from Sc3, who received cascaded training and delivered all modules to their tutor group. In contrast, only those responsible for implementation (Learning Mentors) at Sc2 attended The Guide training; there was therefore no member of the Senior Leadership Team present.

### What adaptations were made and suggested?

Table 3 provides supporting quotes for the different types of adaptations made and suggested to The Guide's training, content and implementation. These are discussed in more detail below in relation to the logistical and philosophical reasons for adaptations.

**Table 3.** Summary of themes relating to what adaptations were made and suggested and the logistical and philosophical reasons

What adaptations were made and suggested	Examples of supporting quotes
**Suggested adaptations to training**	
Planning time and implementation guidance	‘*Because that's such a rarity to go, okay how can we make this work in the schools? And then, almost at the end, feedback across the schools and say, look, these are some of our initial ideas. What do other people think? And kind of bounce off having that interaction.*’ (Sc1, P2)
	‘*So, I would love to have a bit more training possibly, to be able to deliver this content. Because it's quite heavy walking in on a Monday I, just understanding that the lessons have just come in*.’ (Sc3, P10)
**Adaptations made and suggested to content**	
Language	‘*It was changing some of the language so that… not, not to change the factual parts of the language. It was around the language that they can access and understand because what, what we don't want to do is confuse them anymore or make them feel that, you know, we're giving you something that is uncontrollable, or you might not be able to have some control for yourself*.’ (Sc1, P1)
Dropping content	‘*Standard treatments, alternative treatments and complementary. We've dropped it completely. This is an adult page*.’ (Sc1, P1)
	‘*We haven't used the videos because I, we didn't think the videos were that accessible to British students … I'd rather not show a video at all, than show one that the kids don't take seriously*.’ (Sc3, P11)
	‘*The box breathing because that was the bit that some tutors got a bit funny over. You know, they didn't feel quite as confident about that. So, we had left that off*.’ (Sc3, P7)
Emphasising lived experience and student voice	‘*My engagement really was making sure that they understood the importance of being able to speak freely about mental health issues. And as a person who's sought out services in my own personal life, and, you know, vouching for how beneficial it has been in my life, I wanted to speak openly with my tutor group about different types of ways that mental health can kind of manifest itself, and ways that they can cope.*’ (Sc3, P10)
Examples and references	‘*And even the people they were referring to, the students wouldn't necessarily relate to them. So, I asked if there was any information out there about British people in the public eye. So, it was more, I don't know, anglicised I suppose*.’ (Sc1, P3)
	‘*So, for example, the first lesson which includes like, gives them celebrity examples, and we were really keen to find some diverse examples that represented the school*.’ (Sc3, P11)
	‘*If we make that more specific to this area. So, like, what services are available here rather than more widely because, we want them to know what the realistic options are if you go to a GP, what might happen next?*’ (Sc1, P2)
**Adaptations made and suggested to implementation**	
Interactive and student-led approaches	‘*I modified the activity that they gave so it's more a discussion amongst the students. And it was interactive in terms of asking their views on things. So, in that respect, it was a very different kind of presentation, a very different style of delivery as to the one that was presented to us in training*.’ (Sc1, P3)
	‘*Or we split it up so the groups would be doing something and then feeding back to the class as a whole. Creating kind of collages or displays on the wall that, then, students could refer to. So, it was more just thinking about how it wasn't just the teacher delivering that, that the students could be more active*.’ (Sc3, P7)
Lesson plans	‘*Yeah, I mean again it's just it would have been sort of more detailed, more interactive lesson plans in fact I think we would have been happy to go ahead if that had been sort of there from the start*.’ (Sc2, P5)
	‘*So, I think that if there's a way of, if it's rolled out more widely of having like a student-pack, a teacher-pack alongside The Guide so that it's almost easier to like immediately implement, I think that would be really useful*.’ (Sc1, P2)
Support from external organisations and Professionals	‘*But there is a point where I think experts need to come in, to either deliver a training session, or to deliver one of the kind of lessons … they see us with our subject hats on. And so, when we become, you know jack of all trade, it becomes a bit like oh, you can't, they know sometimes we're winging it, you know? And, with something so paramount, so important, you know we wanna do it justice*.’ (Sc3, P10)

Why were adaptations made and suggested?	Examples of supporting quotes
**Logistical reasons**	
Staff capacity and expertise	‘*So that was one of the reasons why, actually, logistically it wasn't very sensible for me to deliver it, because if students are coming out of a lesson, they needed someone they knew they could go to*.’ (Sc3, P7) ‘*So, we decided if we split it up, we'd be able to work better and more effectively on each of the modules rather than one person or us as a group trying to meet which logistically, as I'm sure you know, in the school it's so difficult to get everyone all together*.’ (Sc1, P2) ‘*Our staff simply haven't got the time to change and adapt lessons to make them what they would need to be, to be successful in the, the aims of the project.*’ (Sc2, P6) ‘*The box breathing because that was the bit that some tutors got a bit funny over. You know, they didn't feel quite as confident about that. So, we had left that off*.’ (Sc3, P7)
Timetabling	‘*Because when you pull aside six groups at the same time, the impact it has on the timetable of that because you have to take them out of lessons to do that … Once I started to pencil in a timetable for delivery, it was looking a little cumbersome in terms of covering the lessons for the teachers*.’ (Sc1, P3) ‘*From the school particularly, it would have been from that timetabling of when the lessons were going to be carried out and ensuring you know that everything else was covered.*’ (Sc2, P5)
Accessibility of resources	‘*I think the way it's set out at the moment, the slides and the PDFs, and that I, you couldn't pick those up and teach them, and I think if I had brought this in, and spent money on it, or had like been told will you do this? … I'd be having a nightmare right now because I wouldn't know what to do*.’ (Sc3, P11) ‘*Ensuring lessons were photocopied … that's a genuine question that everyone's concerned about, who pays for photocopying?*’ (Sc2, P5)
**Philosophical reasons**	
Consistency of messages	‘*And when I looked at it. I thought, well, actually, when you've got six people delivering, you can't rely on the consistency of the message coming through. And although we're all good teachers, we all deliver, have a different style. And it's about getting the message across*.’ (Sc1, P3) ‘*I had hoped that myself and two other colleagues would deliver it to the year groups. Because, again, it was that issue of consistency. But logistically it just didn't work, it just wasn't possible. So, what we have done is we've presented to the teams and gone through it with the teams and then they are delivering it. Now, obviously, within that you are going to have better practice than others and that is a reality*.’ (Sc3, P7)
Student characteristics	‘*Looking a bit at diversifying, so some of the videos, I think they pretty much all were white young people which we've got such a diverse mix of young people. To make sure that they didn't think, oh, why am I not represented in it*?’ (Sc1, P2) ‘*Maybe more examples that we could use, say more linked to families and parents or what the children, you know, children, situations with children, could be more, more relevant to them*.’ (Sc3, P9) ‘*There's lots of young people who've not got the literacy levels or the language skills to be able to understand all of that. So just simplifying some of it down but making sure that the content isn't lost.*’ (Sc1, P2)
Reducing stigma and empowering students	‘*Working with those students [with experience of mental health difficulties] and the others I think is a really nice thing to do. I think it gives them, like I say, a little bit more ownership of it and a little bit more understanding and a little bit more empathy*.’ (Sc1, P4) ‘*Doing the posters is quite helpful as well, because the students were then taking on board what they want to say, or what they want to promote*’ (Sc3, P9)
National and local context	‘*I think the British sensibility, you know, that there are just cultural differences and some of those videos you just get that, ugh, no, I don't get this, it is too sincere, it doesn't fit. We are too cynical as a nation*.’ (Sc3, P7) ‘*So rather than it being further removed, they can kind of make sense of it in where they live. Because, even outside of (Borough) it's like, they're like; oh that's, you know, that's such a long way away. So yeah, if we make it too generalised, I think they detach from it a little bit.*’ (Sc1, P2)
Appropriate pedagogic practices	‘*I got the impression from some other schools that they shared that concern that they might be able to do this in Canada, but you just can't stand up and talk at a class. It is not going to wash in terms of when we get observed, and what is expected of us as practitioners. You know you'd be in a lot of trouble if you just, you know, you'd be criticised for it being too teacher led.*’ (Sc3, P7) ‘*We felt that there wasn't really much opportunity for interaction for the pupils to take part in activities, to ask questions to express their views. And our concern was if you know we delivered them like that the pupils could be bored or switch off or they'd misbehave, and it was a really important topic*.’ (Sc2, P5)

### Why were adaptations made and suggested?

Reasons for schools' overall approach to planning and delivery, as well as adaptations made and suggested to The Guide's content and implementation, were organised into logistical and philosophical themes as defined by Moore *et al*. (2013) (see Table 3.).

#### Logistical reasons for adaptations made and suggested

***Staff capacity and expertise:*** Time capacity was a driver for schools in terms of their proactive approaches to adaptation and planning of The Guide implementation and was the primary logistical reason for Sc2 opting not to implement.

‘*So, we decided if we split it up, we'd be able to work better and more effectively on each of the modules rather than one person or us as a group trying to meet which logistically, as I'm sure you know, in the school it's so difficult to get everyone all together.*’ (Sc1, P2)

Staff expertise and previous experience of planning and delivering mental health content was considered by schools when assigning roles, in addition to the relationship between staff and students. The availability of support staff following sessions was also reported to ensure students had someone to go to if they wanted to discuss topics arising or disclose personal difficulties.

‘*So that was one of the reasons why, actually, logistically it wasn't very sensible for me to deliver it, because if students are coming out of a lesson, they needed someone they knew they could go to.*’ (Sc3, P7)

Staff capacity and expertise also led to a range of reported and suggested adaptations to The Guide content and delivery methods, such as structured lesson plans and support from external organisations. Some content was dropped due to a lack of staff confidence, and content relating to lived experience of mental health difficulties was emphasised by those with personal knowledge.

***Timetabling:*** Another logistical challenge for all schools, including Sc2 that decided not to implement The Guide, was timetabling. For Sc1, concerns about pulling students from normal lessons to attend the sessions, and the difficulty of covering staff, informed their implementation approach.

‘*Because when you pull aside six groups at the same time, the impact it has on the timetable of that because you have to take them out of lessons to do that … Once I started to pencil in a timetable for delivery, it was looking a little cumbersome in terms of covering the lessons for the teachers.*’ (Sc1, P3)

For Sc2 and Sc3, that planned to implement as part of the PSHE curriculum, there was an issue of support staff (e.g. Learning Mentors) availability and cover during the timetabled PSHE lessons. This led to the drop out of Sc2 and non-specialist tutors delivering The Guide modules in Sc3.

***Accessibility of resources:*** The suggestion for ready to go, easily accessible and immediately implementable lesson plans also came from school staff finding The Guide website difficult to navigate, and materials time consuming to adapt due to their pdf format. School staff were also unable to play some of the videos due to technical issues, and printing and photocopying was mentioned as a barrier to implementation.

‘*Ensuring lessons were photocopied … that's a genuine question that everyone's concerned about, who pays for photocopying?*’ (Sc2, P5)

#### Philosophical reasons for adaptations made and suggested

***Consistency of messages*:** It was recognised that school staff could have different perspectives on the topics covered in The Guide and adopt different teaching styles. Schools therefore developed approaches to remove personal biases and reduce variation in delivery. By teaching larger groups, Sc1 aimed for students to receive consistent messages while also experiencing a range of staff delivering the modules. Sc3 had hoped to adopt a similar approach, with the two members of staff that attended the training implementing modules. However, due to capacity and timetabling, they instead decided to plan and cascade resources to the class tutors. This approach was viewed as potentially compromising in terms of consistency.

‘*I had hoped that myself and two other colleagues would deliver it to the year groups. Because, again, it was that issue of consistency. But logistically it just didn't work, it just wasn't possible. So, what we have done is we've presented to the teams and gone through it with the teams and then they are delivering it. Now, obviously, within that you are going to have better practice than others and that is a reality.*’ (Sc3, P7)

***Student characteristics*:** Students' characteristics were a driver for adaptations to both content and implementation methods. For example, students' ability and pre-existing knowledge was a reason for dropping content to avoid overloading students with information, and to make messages clear and consistent without too much repetition. Interactive and student-led approaches were also adopted, such as questioning students on their pre-existing knowledge and encouraging them to share their thoughts and experiences. Content relating to different treatment options was described by some as ‘adult’ and deemed too advanced for students. In line with students' ability, language was simplified to make information accessible. Language simplification was both proactive and reactive, for example, in the latter case, a response to the understanding of students speaking English as an additional language. Additional content was also suggested based on gaps in students' pre-existing knowledge (e.g. definition of stigma and examples).

‘*There's lots of young people who've not got the literacy levels or the language skills to be able to understand all of that. So just simplifying some of it down but making sure that the content isn't lost.*’ (Sc1, P2)

Student characteristics in terms of age and ethnic, cultural, and religious background were also considered. For example, content that was not deemed age-appropriate was dropped or adapted, and topics more relevant to young people were suggested for the future (e.g. friendships and parents). Adaptations to examples and references (e.g. examples of celebrities with mental health difficulties and videos) were also made and/or suggested to ensure relatability and that students were represented.

***Reducing stigma and empowering students*:** Some adaptations to content and implementation methods were linked to the overarching aim of reducing stigma and empowering students by normalising experiences and providing strategies for coping and seeking help for themselves and others. For example, in some cases, school staff purposefully adapted language to change the messages in The Guide and de-stigmatise content relating to biomedical explanations of mental illnesses and treatments. The emphasis on lived experience and student voice through interactive and student-led approaches also aimed to reduce the stigma of opening up and talking about mental health and give students' ownership over their learning.

‘*Working with those students [with experience of mental health difficulties] and the others I think is a really nice thing to do. I think it gives them, like I say, a little bit more ownership of it and a little bit more understanding and a little bit more empathy.*’ (Sc1, P4)

***National and local context*:** Adaptations to content were made to ensure the cultural fit of The Guide, with a distinction between ensuring that content was relevant to the local and school context as well as the national context. For example, school staff felt that it was important to provide realistic and accurate information about the accessibility and availability of local and national services and support, suggesting that there was currently a gap in support available in the community and an increased pressure on child and adolescent mental health services. They also wanted to ensure that information aligned with national approaches to diagnosis (e.g. bipolar is rarely diagnosed in childhood). School staff also adapted and suggested national and local prevalence statistics, suggesting that the further removed the content, the less the students would engage with it.

‘*So rather than it being further removed, they can kind of make sense of it in where they live. Because, even outside of (Borough) it's like, they're like; oh that's, you know, that's such a long way away. So yeah, if we make it too generalised, I think they detach from it a little bit.*’ (Sc1, P2)

Similarly, there were examples of videos being dropped due to their lack of cultural fit. For example, Sc2 and Sc3 described The Guide videos as ‘inaccessible’ to British students, and Sc3 did not include them for fear that students would not take them seriously. Language was also anglicised to make it relatable, and examples and references were included that were nationally relevant and fit with the local and school demographic. Finally, in relation to the lack of interactive and student-led delivery methods in The Guide, school staff commented on the possible cultural differences in pedagogic practices between Canada and England and referred to the national standards for school practitioners.

***Appropriate pedagogical practices*:** As previously mentioned under the ‘*National and local context*’ theme, staff used their knowledge of national standards for education practitioners [e.g. Office for Standards in Education (OFSTED)] to adapt implementation. They felt that a predominantly teacher-led and didactic approach would lead to the class not engaging, getting lost, bored and misbehaving. School staff adapted and/or suggested pedagogic practices that were active instead of passive.

‘*We felt that there wasn't really much opportunity for interaction for the pupils to take part in activities, to ask questions to express their views. And our concern was if you know we delivered them like that the pupils could be bored or switch off or they'd misbehave, and it was a really important topic.*’ (Sc2, P5)

Content was dropped to ensure clear and consistent messages, and more interactive and student-led approaches were adopted to increase students' ownership and pride over their learning, help them to apply their experiences to the sessions, and transfer knowledge in the future. There was also recognition across both delivering schools of the sensitive nature of The Guide, and appropriate pedagogy to ensure that an open and non-intimidating environment was created, and a mind-set that would encourage discussions and questions. For example, this included the use of small group work and feeding information from the original Guide mini mags (magazines with facts about different mental health difficulties) back to the class. Workbooks and folders were also reported as a way to make the subject feel valued and important, as well as creating longevity of the project with something for students to look back on and potentially disseminate knowledge at home. Despite some reservations about the cultural appropriateness of The Guide videos, school staff did not reject this mode of delivery for presenting information and other young people's experiences.

## Discussion

In line with recommendations from existing models of cultural adaptation (EVM, CSM) (Bernal *et al*., 1995, 2009; Resnicow *et al*., 1999, 2000), and previous investigations of adaptations of school-based, preventive interventions (Lendrum and Askell-Williams, 2019), the current study found that most adaptations were proactive. Logistical reasons for adaptations such as a lack of preparation and curriculum time aligned with those reported by teachers in studies of the adaptations made to substance misuse prevention (Miller-Day *et al*., 2013) and SEL programmes (Lendrum and Askell-Williams, 2019). Across all schools, staff reported either barriers to being able to send all necessary staff on the training, a lack of opportunity for staff to meet for planning, or difficulties in finding staff cover for the timetabled sessions. It was therefore suggested that more time should be allocated to planning in The Guide training, and that fully developed lesson plans should be provided that can be immediately implemented. Easily accessible lesson plans were also seen as a way to resolve time and technical issues in finding and using specific Guide materials (e.g. videos).

Staff content knowledge and curriculum knowledge, as defined by Shulman's (1986, 1987) categories of teacher knowledge, was also considered by schools when assigning roles, as well as the relationship between staff and students. These codes were combined and included in the logistical theme ‘*Staff Capacity and Expertise*’. For example, the involvement of senior leaders in the delivery of sessions was seen to raise the profile of the topic, the expertise of staff in specialist roles (e.g. SENCO, Mental Health and PSHE Leads) was utilised for the planning and/or implementation, and class tutors were perceived to be well placed to deliver The Guide given their regular and closer contact with students. Support from senior leadership has been identified as essential for the success and sustainability of school-based, mental health and wellbeing initiatives (Askell-Williams, 2017). Both Sc1 and Sc3 were able to send a member of the senior leadership to The Guide training, and at both schools, senior leaders had a role in at least some of the adaptation and planning of The Guide. For Sc2, the fact that it was not possible for a senior leader to attend the training, and that staff assigned to implement The Guide already felt overworked, appeared to contribute to their dropping out of the implementation.

The aims of The Guide were generally accepted. However, the importance of adequate training was emphasised in the current study with some staff from Sc3, who received only a cascaded version of the training within school, feeling underprepared. Despite an attempt to carefully balance the expertise and availability of staff, there were examples of content being dropped that staff did not feel confident delivering, and concerns were raised about schools' capacity to deal with potential disclosures following The Guide sessions. In Lendrum and Askell-Williams’ (2019) study of teachers' adaptations to the Promoting Alternative Thinking Strategies (PATHS) Program, SEL was perceived as a general practice that did not require discrete subject knowledge. In contrast, the content in The Guide was perceived to require professional subject knowledge that should be delivered by staff within the school with the most expertise in mental health, and with continued support from external mental health professionals. These findings align with barriers previously identified for delivering school-based mental health provision such as limited guidance, staff capacity and consultation and support from external mental health professionals (Vostanis *et al*., 2013; Patalay *et al*., 2016; Sharpe *et al*., 2016; Shelemy *et al*., 2019; Mansfield *et al*., 2021).

Taken together, the logistical reasons for adaptations speak to the fact that up until recently, mental health education was not compulsory in English schools (Department for Education, 2019). Schools adopted different approaches to adaptation, planning and implementation due to staff expertise, availability and timetabling issues. For example, the range of roles held by staff, and the different approaches to timetabling sessions, shows a lack of consistent staffing (e.g. Mental Health Leads and Support Staff) and allocated time for mental health initiatives. There was an attempt within schools to provide consistent messages to students, acknowledging not only staff members' different teaching styles but also the influence of their knowledge, beliefs and experiences relating to mental health. School staff reported an awareness of different professional mental health discourses (Zeeman and Simons, 2011), and the effect of inconsistent messages and predominantly biomedical explanations on desired stigma reduction.

Examples of both deep and surface-level adaptations were reported, as defined by existing models of cultural adaptation (EVM, CSM) (Bernal *et al*., 1995, 2009; Resnicow *et al*., 1999, 2000). However, there was some overlap between these codes and the decision was taken to organise themes based on the aspects of content referred to. For example, school staff reported and suggested adaptations to surface-level components of The Guide content, like language, and examples and references such as the people represented in videos, and reference to local organisations. Philosophical reasons included student characteristics such as ability, as well as characteristics of the national and local context (e.g. availability of services). Adaptations to language were also used to change key messages in The Guide, which was perceived to be a deep-level conceptual change to align with school staffs' beliefs about what messages would most likely produce positive outcomes. Similarly, emphasis on lived experience and student voice was associated with achieving the core outcomes of reducing stigma and empowering students to seek help for themselves and others. Although these were the original aims of The Guide, a process of dropping, refining and adding content to achieve these aims was perceived to be necessary.

The majority of adaptations made and suggested were to The Guide's implementation methods. These predominantly consisted of creating interactive and student-led approaches which were driven by student characteristics, national standards for appropriate pedagogical practices, and the aim of reducing stigma and empowering students. These philosophical reasons for adaptations align with Shulman's (1986, 1987) categories of teachers' knowledge found to most commonly inform adaptations made to the PATHS curriculum in the UK (Lendrum and Askell-Williams, 2019), namely, ‘knowledge of learners and their characteristics’ and ‘pedagogic content knowledge’. Staff applied their general pedagogic knowledge as well as an understanding of the kinds of pedagogies appropriate for covering mental health topics and reported reducing the PowerPoint slides and replacing them with interactive and student-led activities, discussions and group work. The hope was that students would take more ownership over their learning, apply their knowledge to their own experiences, and feel a sense of reduced stigma in discussing mental health and seeking help for themselves and others. They reported a potential cultural mismatch between students' learning styles in Canada and England (Castro *et al*., 2004), questioning the ability of Canadian students to behave and listen to a teacher deliver a long PowerPoint presentation.

### Strengths and limitations

Interviews took place mid to late delivery, meaning that schools had not yet completed implementing The Guide. This may have contributed to fewer reactive adaptations being reported by school staff. Although the current study had a small sample size, it provides an in-depth exploration of the different approaches of three schools that were allocated to deliver The Guide as part of the EfW feasibility study. Rich accounts from a small sample can be seen as a strength of qualitative research (Smith, 2018). Instead of applying statistical-probabilistic generalisability, the current study conducted a hybrid thematic analysis to explore analytical generalisability, i.e. conceptual, or theoretical generalisations relating to cultural adaptations made to school-based interventions. Given the current study also presents schools' reflections on their approaches to implementing The Guide, this offers opportunities for naturalistic and transferable generalisability, in which the reader may identify more with a particular school's experiences and apply this to their own school context (Smith, 2018). Of course, it is important to note that the schools involved in the current study, at least at the point of expressing interest in the EfW programme, felt able to implement a set of MHL lessons. This self-selection indicates a priority afforded to improving students' MHL. This does not mean, however, that other schools cannot learn from their experiences when considering implementing similar interventions in the future.

### Implications

Given that most of the adaptations made and suggested to The Guide related to the process of implementation within school and to the teaching delivery methods, it could be concluded that school staff felt the content was, for the most part, culturally appropriate. However, it could also point to the fact that overcoming logistical barriers and pedagogical standards may have been the bigger priority for schools and that, with greater capacity, school staff would have liked to adapt the content further. If schools that are motivated and better equipped to deliver these types of interventions make adaptations due to logistical constraints, there are clear implications for the future uptake of such approaches by schools across England.

The current study therefore adds insights specific to MHL interventions and potential barriers for mental health education more generally in the English school context. For example, schools must be properly resourced to enable them to release all appropriate staff on relevant training, and the training should provide designated time for the planning of implementation. As is indicated by the current study, the success of mental health education interventions in England relies on building better joint working approaches between external mental health professionals and school staff. Coaching models could be one solution for implementing MHL interventions in the future, whereby school staff receive ongoing training and support to deliver mental health content (Ashworth *et al*., 2018).

Informed by data analysed in the current study, informal feedback from non-case study schools, interviews and focus groups with young people that received the intervention, and young advisors from a lived experience in mental health consultancy organisation, The Guide was adapted for trial in England. Adaptations were made by the training and development team at the Anna Freud National Centre for Children and Families, a registered mental health charity. Staff included trained teachers with expertise in school-based mental health programmes and child and adolescent clinical psychologists. Six ready-made lesson plans were produced to reduce preparation time. Each lesson was made available digitally and included a lesson plan with objectives, PowerPoint slides, activities, video links and teacher guidance. A signposting poster was also made available to schools including national-level information and support services (e.g. Youth Wellbeing Directory), quotes from students involved in the feasibility study, and space for school staff to include support staff in school (e.g. Pastoral Lead) and local organisations and services. A reduction of content was agreed with the intervention developer to incorporate more interactive approaches. The newly developed Guide training incorporated ways for teachers to facilitate discussion, debate and encourage criticality amongst students. This was accompanied by information on the different professional discourses around mental health in England, and an acknowledgement of the biomedical influence on the original Canadian version of The Guide. There was also more information relating to managing potential disclosures that could arise in and following The Guide sessions.

Given that cultural adaptation is inevitable, understanding the ‘active ingredients’ while applying a realist approach to the mechanisms of change for school-based MHL interventions is key. In the current study, school staff adapted some of The Guide's content to ensure a de-stigmatising approach; more research is needed to understand exactly what knowledge and skills must be acquired to promote young people's mental health (Cairns and Rossetto, 2019). To ensure that a ‘fidelity *with* adaptation’ approach recommended by Lendrum and Askell-Williams (2019) was accounted for in the full trial of The Guide, a full process and implementation evaluation was conducted alongside the AWARE efficacy trial as detailed in the study protocol (Hayes *et al*., 2019). This will enable the analysis to identify the impact of different dimensions of implementation (e.g. fidelity, dosage and adaptation), on outcomes.

## Conclusion

In its original format, The Guide is exactly what it says it is: a curriculum *guide* designed for global application. School staff valued the ‘*one stop-shop*’ approach, however, as described by Castro *et al*. (2010), there was a tension between wanting immediately implementable lesson plans that could be delivered with fidelity by any member of school staff, and the flexibility to adapt lessons to fit the characteristics of their students and the local context. The aim of the EfW feasibility study was to adapt the imported interventions for the English school context to then evaluate their efficacy in a clustered RCT. Data from the current study indicate that school staff believe that adapting content for the English school context goes only part of the way to ensuring the aims of the intervention are met. Suggestions for greater involvement of teachers in the design of lesson plans support Lendrum and Askell-Williams’ (2019) recommendation that interventions should be developed with the input of teachers' knowledge about best pedagogic practices, and the level of flexibility necessary to accommodate contextual factors and students' characteristics. Creating space for local adaptation while maintaining clarity on the core components of an intervention can help to reduce tensions experienced by those delivering and will increase the likelihood of successful implementation.
